# Transient response improvement of predictive control under time domain constraints

**DOI:** 10.1016/j.heliyon.2024.e40260

**Published:** 2024-11-12

**Authors:** Khelifa Khelifi Otmane, Taieb Bessaad

**Affiliations:** a Blida University, Department of Automatic and Electrotechnic, B.P 270 Route de Soumâa, Blida, Algeria; b Electrotechnical, Hassiba Benbouali University, LGEER Laboratory, Chlef, Algeria

**Keywords:** Convex optimization, Generalized predictive control, Input constraints, Overshoot, RST controller, Settling time, Settling error

## Abstract

In this paper, a novel predictive controller design approach within augmented structure base on convex optimization of an extra parameter is proposed in order to improve the transient response of discrete-time linear systems subject to input constraints. This methodology starts with the conception of an initial stabilizing predictive controller which ensures a fast closed loop response to the detriment of the other response specifications indexes (settling time, settling error, overshoot and control signal). Then, this controller is redesigned off-line using linear programming solver to meet a certain transient response specifications of the closed loop system determined before the design as time domain constraints. The resulting controlled output will track the reference as fast and as smooth as possible with a low energy consumption. Two examples are given to show the effectiveness of the developed controller.

## 1 Introduction

The Generalized Predictive Control (GPC) [1] is an advanced technique of process control. It has been implemented successfully in various industrial applications [[2], [3], [4]], it is due to its optimality robustness, and ability to face uncertainty. In absence of system constraints, it is possible to obtain the optimal solution in an analytic form and the GPC control law is pre-calculated off-line which leads to a **R**eference **S**ignal **T**racking (RST) controller. It has a two **D**egree **O**f **F**reedom structure (2DOF) and comprises of three polynomials namely R, S and T. The RST configuration has become very useful in industrial applications because of their structural simplicity and easy implementation [5]. Moreover, with this configuration, the tracking and regulation characteristics of a closed-loop system can be handled independently [6].

Despite its qualities, GPC is deficient when severe specifications are required and it needs to be improved especially in terms of transient response behavior. Indeed, when we must consider a number of issues including specifications on time-domain signals such as settling time, actuator amplitude limits and output signal overshoot, GPC_RST (GPC with RST structure) controller cannot achieve concurrently these conflicting performance goals. In fact, small overshoot and quick response are desirable in many control systems. Yet, a fast dynamic response to set-point changes often causes a large overshoot and longer settling time. Therefore, there is a trade-off between these specifications particularly for systems whose control input is limited in magnitude. As a consequence, the selection of the GPC parameters (prediction horizons, control horizon and control weighting factor) becomes insufficient method to manage the compromise between all these specifications.

The problem of obtaining good transient response performance has been addressed in many control systems using various techniques [7,8,9,10,11,and^12^]]. For 2DoF controller design, some procedures can be found in the literature aiming at the design of a suitable feed-forward or pre-filtering control action intended to improve the reference tracking performance of an existing feedback controller [13,14,and^15^]]. These approaches maintain the performance and design principles of the original controller by adding complementary parts to an existing controller structure. Besides these control strategies, that are typically suited for a continuous-time context, there are only a few methods available in the literature that can synthesize controllers incorporating time-domain specifications in the discrete-time setting [16,17,18,and19]], all of which are for non-GPC scheme. As for GPC under RST framework, methodologies have been developed to improve qualities of GPC_RST controllers, introducing for that purpose extra parameters, such as the Youla parameters [20,and^21^]], these methods are off-line procedure based on an initial RST controller that is robustified via the Youla parametrisation toward model uncertainties at a second level. This parameterisatlon allows formulating frequency and temporal constraints as an optimization problem which is solved through a linear programming structure under inequality constraints.

The contribution of this paper is to propose an extending and simple design scheme to improve the transient performance of the GPC_RST controller. This is achieved by shaping the transient response of the closed-loop system according to the performance specifications determined before the design. Our procedure is based on two-step design paradigm. First, we design a basic GPC_RST controller for a nominal plant to provide the better closed loop performance with quick response to input unit-step focusing on the standard generalized predictive control. In the second step, the feed-forward term is adjusted to achieve the required tracking specifications. In fact, we will introduce an extra parameter *F* in parallel with the tracking polynomial *T*, where the polynomials R, S remains fixed assuming the feedback part is already in place thanks to the initial controller. We define this parameter as a finite impulse response (FIR) filter and design it by formulating the temporal constraints as a convex optimization problem. Thus, the new RST controller preserves the same RST structure with better results in terms of transient response behavior while the input control bounds will be respected.

The rest of the paper is organized as follows. Section II reminds the main steps leading to the GPC controller in RST formalism. Section III gives the basic steps leading to the improved GPC controller. Section IV provides the application of this control strategy on two examples. Section V presents some conclusion.

## 2 GPC controller design

Based on the CARIMA (Controlled Autoregressive Integrated Moving Average) model used by the Generalized Predictive Control (GPC) [22] we describe the process as:

(1) $$A\left(q^{-1}\right)y\left(t\right)=B\left(q^{-1}\right)u\left(t\right)+\frac{\xi \left(t\right)}{\Delta \left(q^{-1}\right)}$$

Where *u(t)* and *y(t)* are the control and output sequences of the process, and ξ(t) is a zero-mean white noise. *A* and *B* are polynomials in backward shift operator $q^{-1}$ in the form:

$$A\left(q^{-1}\right)=1+{a_{1}q}^{-1}+a_{2}q^{-2}+\ldots +a_{na}q^{-na}$$

$$B\left(q^{-1}\right)=b_{0}+{b_{1}q}^{-1}+b_{2}q^{-2}+\ldots +b_{nb}q^{-nb}$$

And $\Delta \left(q^{-1}\right)=1-q^{-1}$ the difference operator

The *j-*step ahead prediction over the costing horizons $N_{1}\leq j\leq N_{2}$ is given by:

(2) $$y\left(t+j\right)=\underset{\mathrm{free}\;\mathrm{response}}{\underbrace{F_{j}\left(q^{-1}\right)y\left(t\right)+H_{j}\left(q^{-1}\right)\Delta u\left(t-1\right)}}+\underset{\mathrm{forced}\;\mathrm{response}}{\underbrace{G_{j}\left(q^{-1}\right)\Delta u\left(t+j-1\right)+J_{j}\left(q^{-1}\right)\xi \left(t+j\right)}}$$

With $G_{j}$ representing the future, $F_{j},H_{j}$ corresponding to the present and the past, and $J_{j}$ linked to disturbances.

By combining the CARIMA model equation (1) with the predictor equation (2), we obtain the next system equations

(3) $$\left\{ \begin{array}{c} A\left(q^{-1}\right)\Delta \left(q^{-1}\right)y\left(t+j\right)=B\left(q^{-1}\right)\Delta u\left(t+j-1\right)+\xi \left(t+j\right)\;\\ \left[1-q^{-j}F_{j}\left(q^{-1}\right)\right]y\left(t+j\right)=\left[G_{j}\left(q^{-1}\right)+q^{-j}H_{j}\left(q^{-1}\right)\right]\Delta u\left(t+j-1\right)+J_{j}\left(q^{-1}\right)\xi \left(t+j\right) \end{array}\right.$$

Then ${\;F}_{j},G_{j}{,H}_{j}{,J}_{j}$ are obtained by solving the following Diophantine equations:

(4) $$\left\{ \begin{array}{c} \Delta \left(q^{-1}\right)A\left(q^{-1}\right)J_{j}\left(q^{-1}\right)+q^{-j}F_{j}\left(q^{-1}\right)=1\\ G_{j}\left(q^{-1}\right)+q^{-j}H_{j}\left(q^{-1}\right)=B\left(q^{-1}\right)J_{j}\left(q^{-1}\right) \end{array}\right.$$

The optimal predictor deduced from the consideration that the best estimation of disturbing signal in the future is equal to its average (equal to 0 in case of zero-mean white noise), takes the form:

(5) $$\hat{y}\left(t+j\right)=F_{j}\left(q^{-1}\right)y\left(t\right)+G_{j}\left(q^{-1}\right)\Delta u\left(t+j-1\right)+H_{j}\left(q^{-1}\right)\Delta u\left(t-1\right)$$

With

$$\begin{array}{c} \text{degree}\;\text{of}\;\left[G_{j}\left(q^{-1}\right)\right]=j-1\;\\ \text{degree}\;\text{of}\;\left[F_{j}\left(q^{-1}\right)\right]=\text{degree}\;\text{of}\;\left[A\left(q^{-1}\right)\right]\;\\ \text{degree}\;\text{of}\;\left[H_{j}\left(q^{-1}\right)\right]=\text{degree}\;\text{of}\;\left[B\left(q^{-1}\right)\right]-1 \end{array}$$

To achieve optimal command values, the GPC uses a quadratic cost function defined as:

(6) $$J\left(N_{1},N_{2}\right)=\sum_{j=N_{1}}^{N_{2}}{\left[\hat{y}\left(t+j\right)-w\left(t+j\right)\right]}^{2}+\lambda \sum_{j=1}^{N_{u}}{\left[u\left(t+j-1\right)\right]}^{2}$$

$$\Delta u\left(t+j\right)=0\;\text{for}\;j\geq N_{u}$$

Where $N_{1}$ and $N_{2}$ define the output prediction horizons, and $N_{u}$ the control horizon. λ is the control weighting factor, *w* the set-point value, $\hat{y}$ is the predicted output value, obtained solving Diophantine equation, and *u* is the control signal.

**Fig. 1:**
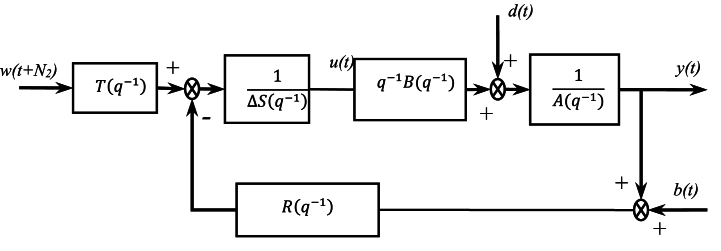
GPC Equivalent polynomial RST controller.

The receding horizon principle assumes that only the first value of optimal control series resulting from the optimization of δJ/δu is applied, so that for the next step this procedure is repeated. Thus, the design has been performed adjusting $N_{1},{\;N}_{2}{,N}_{u},\lambda$ parameters to satisfy the required input/output behavior: fastest response consistent with stability requirements. This control strategy leads to a 2-DOF RST controller (Fig. 1) implemented through a difference equation:

(7) $$\Delta \left(q^{-1}\right)S\left(q^{-1}\right)u\left(t\right)=-R\left(q^{-1}\right)y\left(t\right)+T\left(q\right)w\left(t\right)$$

Where, *b(t)* and *d(t)* are the disturbances.

## 3 Problem formulation and design steps

It can be shown that the GPC_RST controller shown in (Fig. 1) contains a feedback and feedforward terms allowing, respectively, the achievement of two levels of performance in regulation and tracking. In our further developments, we will assume that the initial GPC_RST controller has been performed with $N_{1},N_{2},N_{u},\lambda$ adjusted to satisfy certain closed loop performance with fastest response by allowing a certain amount of settling error and overshoot. We further consider the improvement on the transient behavior by using a new GPC_RST control system structure.

**Fig. 2:**
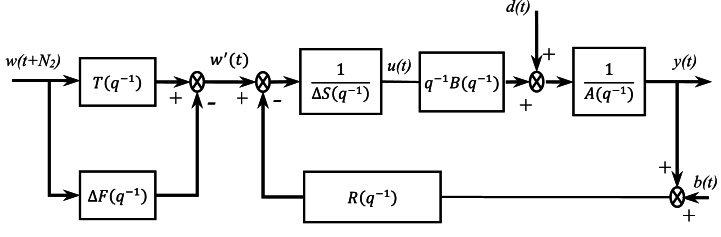
The proposed control structure.

The control structure under consideration is shown in Fig. 2, and it is given by polynomials $R\left(q^{-1}\right)$, $S\left(q^{-1}\right)$, $T\left(q^{-1}\right)$ and $F\left(q^{-1}\right)$, which can be expressed as:

(8) $$\left\{ \begin{array}{l} R\left(q^{-1}\right)=r_{0}+{r_{1}q}^{-1}+r_{2}q^{-2}+\ldots +r_{nr}q^{-nr}\\ S\left(q^{-1}\right)=s_{0}+{{sb}_{1}q}^{-1}+s_{2}q^{-2}+\ldots +s_{ns}q^{-ns}\\ T\left(q^{-1}\right)=t_{0}+{t_{1}q}^{-1}+t_{2}q^{-2}+\ldots +t_{nt}q^{-nt}\\ F\left(q^{-1}\right)=f_{0}+{f_{1}q}^{-1}+f_{2}q^{-2}+\ldots +f_{nf}q^{-nf} \end{array}\right.$$

The $R\left(q^{-1}\right)$, $S\left(q^{-1}\right)$ and $T\left(q^{-1}\right)$ polynomials are the same obtained by the initial controller, and $\Delta F\left(q^{-1}\right)$ a stable extra feedforward pre-filter placed in parallel connection with the tracking polynomial $T\left(q^{-1}\right)$. This extra feedforward pre-filter is tuned to provide the better tracking performance in terms of speed of response, settling time, overshoot and eventually, reducing the excessive controller output change on reference changes. Note that $\Delta F\left(q^{-1}\right)$ has no implications on the general stability of the resulting control system (as long as the added block is itself stable).

Following the controller structure given in Fig. 2, the closed-loop transfer function of the overall system can be represented from the reference w to the controlled variable y and from the reference w to the control signal u are, respectively, given by:

(9) $$G_{yw}=\frac{y}{w}=\frac{Tq^{-1}B}{P_{c}}-\frac{q^{-1}B}{P_{c}}\Delta F$$

(10) $$G_{uw}=\frac{u}{w}=\frac{TA}{P_{c}}-\frac{A}{P_{c}}\Delta F$$

$P_{c}=\Delta A{S+q}^{-1}BR$ is the characteristic polynomial of the closed loop obtained with the initial controller. These transfer functions are linearly parametrized by F [23]. In the steady state (for q=1), the output *y* must tends towards the reference *w*. this justifies the presence of the integrator term Δ in the second part of the relation (9), such as the first part naturally possess a unit gain with the initial structure of the GPC_RST controller. So we can write:

(11) $$\frac{y\left(t\right)}{w\left(t\right)}={\frac{Tq^{-1}B}{P_{c}}-\frac{q^{-1}B}{P_{c}}\Delta F|}_{q=1}=1$$

The relation (9) can be written as

(12) $$G_{yw}=T_{1}+T_{2}F$$

Also we can write

(13) $$\frac{y\left(t\right)}{w\left(t\right)}=T_{1}+T_{2}\sum_{i=0}^{n_{f}}f_{i}q^{-i}$$

Next, we suppose that the system is excited with a step reference input w.

Then, the output response y expression is obtained as

(14) $$y\left(t\right)=w\left(t\right)T_{1}+w\left(t\right)T_{2}\left(f_{0}+f_{1}q^{-1}+\ldots +f_{n_{f}}q^{-n_{f}}\right)$$

We note

(15) $$y_{1}\left(t\right)={wT}_{1}$$

And

(16) $$y_{2}\left(t\right)=w\left(t\right)T_{2}\sum_{i=0}^{n_{f}}q^{-i}$$

The output response expression can then be rewritten in the vector representation:

(17) $$y\left(t\right)=y_{1}\left(t\right)+\left[\begin{array}{ccc} y_{20}\left(t\right) & y_{21}\left(t\right) & \begin{array}{cc} \cdots & y_{2n_{f}}\left(t\right) \end{array} \end{array}\right]\left[\begin{array}{c} f_{0}\\ \vdots \\ f_{n_{f}} \end{array}\right]$$

**Fig. 3:**
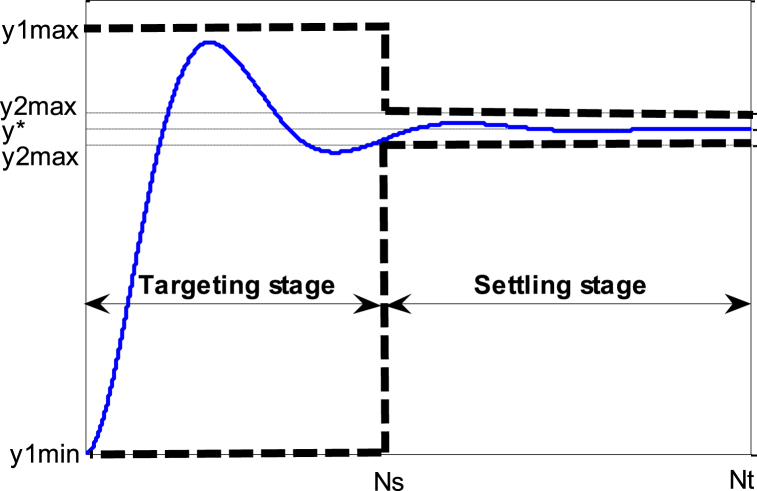
Definition of the output constraints.

The problem aims at finding F that improves the reference tracking capability and mitigates the overshoot without compromising the speed of the system response. To achieve this goal, the system output y(t) will subject to two different amplitude constraints as depicted in Fig. 3 reflecting these three conditions:

- The overshoot must be smaller than a desired value;
- Settling time must be shorter than a desired value;
- Settling error must be within expected bounds.

By considering the $N_{t}+1$ first values of the response y(t), we impose the first time domain constraints corresponds to the overshoot constraints during the targeting stage (e.g. from 0 to $N_{s}$ where $N_{s}$ denotes the number of samples to reach the target correspond to the settling time.

(18) $$y_{1\;\min}\left(t\right)\leq y\left(t\right)\leq y_{1\;\max}\left(t\right)\;\text{for}\;{\;t=t}_{0},t_{1},\ldots ,t_{N_{s}}$$

Also, we can write

(19) $$y\left(t\right)-y_{1\;\max}\left(t\right)\leq 0-y\left(t\right)+y_{1\;\min}\left(t\right)\leq 0\}\;\text{for}\;{\;t=t}_{0},t_{1},\ldots ,t_{N_{s}}$$

Similarly, we impose the second condition during the settling stage (e.g. from $N_{s}\;\text{to}\;N_{t}$),

(20) $$y_{2\;\min}\left(t\right)\leq y\left(t\right)\leq y_{2\;\max}\left(t\right)\;\text{for}\;{\;t=t}_{N_{s}},\ldots ,t_{N_{t}}$$

Also, we can write

(21) $$y\left(t\right)-y_{2\;\max}\left(t\right)\leq 0-y\left(t\right)+y_{2\;\min}\left(t\right)\leq 0\}\;\text{for}\;{\;t=t}_{N_{s}},\ldots ,t_{N_{t}}$$

These inequalities lead to the cost minimization under inequality constraints. Let β the decision variable to be minimized. So, the cost minimization can be written as follows:

(22) $$\min_{A_{1}X-B_{1}\leq 0}CX$$

Where

$$A_{1}={\left[\begin{array}{ccccc} 0 & y_{20}\left(t_{0}\right) & y_{21}\left(t_{0}\right) & \cdots & y_{2n_{q}}\left(t_{0}\right)\\ 0 & y_{20}\left(t_{1}\right) & y_{21}\left(t_{1}\right) & \cdots & y_{2n_{q}}\left(t_{1}\right)\\ \vdots & \vdots & \vdots & \cdots & \cdots \\ 0 & y_{20}\left(t_{N_{s}}\right) & y_{21}\left(t_{N_{s}}\right) & \cdots & y_{2n_{q}}\left(t_{N_{s}}\right)\\ 0 & y_{20}\left(t_{N_{s}+1}\right) & y_{21}\left(t_{N_{s}+1}\right) & \cdots & y_{2n_{q}}\left(t_{N_{s}+1}\right)\\ \vdots & \vdots & \vdots & \ddots & \vdots \\ 0 & y_{20}\left(t_{N_{t}}\right) & y_{21}\left(t_{N_{t}}\right) & \cdots & y_{2n_{q}}\left(t_{N_{t}}\right)\\ 0 & -y_{20}\left(t_{0}\right) & -y_{21}\left(t_{0}\right) & \cdots & {-y}_{2n_{q}}\left(t_{0}\right)\\ \vdots & -y_{20}\left(t_{1}\right) & -y_{21}\left(t_{1}\right) & \cdots & -y_{2n_{q}}\left(t_{1}\right)\\ \vdots & \vdots & \vdots & \cdots & \vdots \\ 0 & -y_{20}\left(t_{N_{s}}\right) & -y_{21}\left(t_{N_{s}}\right) & \cdots & {-y}_{2n_{q}}\left(t_{N_{s}}\right)\\ 0 & -y_{20}\left(t_{N_{s}+1}\right) & -y_{21}\left(t_{N_{s}+1}\right) & \cdots & {-y}_{2n_{q}}\left(t_{N_{s}+1}\right)\\ \vdots & \vdots & \vdots & \ddots & \vdots \\ 0 & -y_{20}\left(t_{N_{t}}\right) & -y_{21}\left(t_{N_{t}}\right) & \cdots & {-y}_{2n_{q}}\left(t_{N_{t}}\right) \end{array}\right]}_{\left({2N}_{t}+2\right)\times \left(n_{q}+1\right)}$$

$$B_{2}={\left[\begin{array}{c} 0\\ y_{max}\left(t_{0}\right)-y_{1}\left(t_{0}\right)\\ \vdots \\ y_{\max}\left(t_{N_{s}}\right)-y_{1}\left(t_{N_{s}}\right)\\ \vdots \\ \begin{array}{c} y_{\max}\left(t_{N_{t}}\right)-y_{1}\left(t_{N_{t}}\right)\\ {-y}_{\min}\left(t_{0}\right)+y_{1}\left(t_{0}\right)\\ \begin{array}{c} \vdots \\ {-y}_{\min}\left(t_{N_{s}}\right)+y_{1}\left(t_{N_{s}}\right)\\ \vdots \\ {-y}_{\min}\left(t_{N_{t}}\right)+y_{1}\left(t_{N_{t}}\right) \end{array} \end{array} \end{array}\right]}_{\left({2N}_{t}+2\right)\times 1},X={\left[\begin{array}{c} \beta \\ f_{0}\\ \cdots \\ f_{n_{f}} \end{array}\right]}_{\left(n_{f}+1\right)\times 1}$$

$$C={\left[\begin{array}{cccc} 1 & 0 & \cdots & 0 \end{array}\right]}_{1\times \left(n_{q}+2\right)}$$

At this stage, we intend to consider the energy consumption reduction by specifying constraints on the control signal u. We define amplitude constraints on the control signal as:

(23) $$u_{\min}\leq u\leq u_{\max}$$

equation (10) can be rewritten as:

(24) $$\frac{u\left(t\right)}{w\left(t\right)}=T_{1}^{\prime }+T_{2}^{\prime }\sum_{i=0}^{n_{f}}f_{i}q^{-i}$$

The response u(t) to the reference input w(t) expression is obtained as

(25) $$u\left(t\right)=w\left(t\right)\;T_{1}^{\prime }+w\left(t\right)T_{2}^{\prime }\left(f_{0}+f_{1}q^{-1}+\ldots +f_{n_{f}}q^{-n_{f}}\right)$$

We note

(26) $$u_{1}\left(t\right)=wT_{1}^{\prime }$$

and

(27) $$u_{2}\left(t\right)=w\left(t\right)T_{2}^{\prime }\sum_{i=0}^{n_{f}}q^{-i}$$

The expression (25) can be rewritten in the vector representation:

(28) $$u\left(t\right)=u_{1}\left(t\right)+\left[\begin{array}{ccc} u_{20}\left(t\right) & u_{21}\left(t\right) & \begin{array}{cc} \cdots & u_{2n_{f}}\left(t\right) \end{array} \end{array}\right]\left[\begin{array}{c} f_{0}\\ \vdots \\ f_{n_{f}} \end{array}\right]$$

In the same manner as in the previous development, only the $N_{t}+1$ first samples of the response u(t) are considered. The constraints (23) can be expressed as

(29) $$u\left(t\right)-u_{\max}\left(t\right)\leq 0-u\left(t\right)+u_{\min}\left(t\right)\leq 0\}\;\text{for}\;t=0,\ldots ,t_{N_{t}}$$

The constraints (29) can be equivalently written as a linear constraint

(30) $$A_{2}X-B_{2}\leq 0$$

With

$$A_{2}={\left[\begin{array}{ccccc} 0 & u_{20}\left(t_{0}\right) & u_{21}\left(t_{0}\right) & \cdots & u_{2n_{q}}\left(t_{0}\right)\\ 0 & u_{20}\left(t_{1}\right) & u_{21}\left(t_{1}\right) & \cdots & u_{2n_{q}}\left(t_{1}\right)\\ \vdots & \vdots & \vdots & \cdots & \cdots \\ 0 & u_{20}\left(t_{N_{t}}\right) & u_{21}\left(t_{N_{t}}\right) & \cdots & u_{2n_{q}}\left(t_{N_{t}}\right)\\ 0 & -u_{20}\left(t_{0}\right) & -u_{21}\left(t_{0}\right) & \ddots & {-u}_{2n_{q}}\left(t_{0}\right)\\ 0 & -u_{20}\left(t_{1}\right) & -u_{21}\left(t_{1}\right) & \cdots & -u_{2n_{q}}\left(t_{1}\right)\\ \vdots & \vdots & \vdots & \cdots & \cdots \\ 0 & -u_{20}\left(t_{N_{t}}\right) & -u_{21}\left(t_{N_{t}}\right) & \cdots & {-u}_{2n_{q}}\left(t_{N_{t}}\right) \end{array}\right]}_{\left({2N}_{t}+2\right)\times \left(n_{q}+2\right)}$$

$$B_{2}={\left[\begin{array}{c} u_{\max}\left(t_{0}\right)-u_{1}\left(t_{0}\right)\\ \vdots \\ \begin{array}{c} u_{\max}\left(t_{N_{t}}\right)-u_{1}\left(t_{N_{t}}\right)\\ {-u}_{\min}\left(t_{0}\right)+u_{1}\left(t_{0}\right)\\ \begin{array}{c} \vdots \\ {-u}_{\min}\left(t_{N_{t}}\right)+u_{1}\left(t_{N_{t}}\right) \end{array} \end{array} \end{array}\right]}_{\left({2N}_{t}+2\right)\times 1}$$

Which must be added to the constraints defined by (22)

(31) $$\begin{array}{cc} \min & CX\\ \begin{array}{c} A_{1}X-B_{1}\leq 0\\ A_{2}X-B_{2}\leq 0 \end{array} & \end{array}$$

Solving the resulting linear programming (LP) problem (31) provides the coefficients $\left[\begin{array}{ccc} f_{0} & f_{1} & \begin{array}{cc} \cdots & f_{n_{f}} \end{array} \end{array}\right]$. The command *linprog* of MATLAB will be used as LP solver:

X=linprog(C,[A1;A2],[B1;B2]);

$$F={\left[X\left(1:\text{length}\left(X\right)-1,:\right)\right]}^{\prime }\text{;}$$

If resolution problems due to numerically ill-conditioned minimization appear, a solution consists of either enlarging the order of the polynomial F or relaxing the time-domain constraints. But a high order polynomial generates an expensive controller in terms of execution and time memory space. To avoid this problem, the polynomial F can be approximated by a small-order transfer function.

This approximation can be obtained by the infinite impulse response (IIR) filter synthesis method proposed in Refs. [19,20].

The main advantage of this method is that all the results are based on off-line calculations offering qualitative information prior to the effective implementation.

## 4 Numerical results

In this section, we apply our proposed strategy to some numerical examples. We performed simulations with MATLAB software in the discrete time.

### 4.1 Example 1: unstable 2nd order process

In this example, we use an example reported in Ref. [24], the process in continuous time is given by

$$G\left(s\right)=\frac{s+0.5}{s\left(s-2\right)}$$

We use Euler method with sampling period ${\;T}_{s}=0.1s$. The obtained discrete time model is

$$G\left(q^{-1}\right)=q^{-1}\frac{0.1134-0.1078q^{-1}}{1+{-2.2214q}^{-1}+1.2214q^{-2}}$$

The control objective is to let output response track a step reference from 0 to 1 fastly with minimal settling time and without overshoot. As mentioned in section III, this goal cannot be reached by the conventional GPC predictive control. Below, we will demonstrate that this is possible by applying the proposed methodology developed in section III.

As a first step, an initial GPC_RST controller has been first designed to achieve a fast response without focusing on the other transient performances indexes e.g. settling time, overshoot and control effort. The following tuning parameters are chosen ${\;N}_{1}=2,N_{2}=6,N_{u}=1$. The simulation shows that the initial controller provides a fast rising time of 1s, with an overshoot of 122 % and very longer settling time about 6.3s, the control effort is also high.

In the second step, we will introduce our proposed approach to reduce the settling time and mitigate the overshoot such that the input control constraints will not violated while keeping the rising time closest as possible to that obtained by the initial controller. Consequently, the optimization problem of equation (28) has to be solved satisfying these conditions:

$$\text{Zero}\;\text{overshoot}\;\text{and}\;\text{zero}\;\text{undershoot}\;\to \;y_{1\;\max}=1,y_{1\;\min}=0$$

$$\text{Short}\;\text{settling}\;\text{time}\;\to \;N_{s}T_{s}=2s$$

$$\text{Minimal}\;\text{settling}\;\text{error}\;\to \;y_{2\;\max}=1.01,y_{2\;\min}=0.99$$

Controlsignalbounded→−1.2≤u(t)≤1.2

The research of the F parameter has been achieved with *40-*order polynomial and with $N_{t}=200$ samples equivalent to 10s. The choice of the order of the polynomial F is based on an empirical process such that a low order leads to a restriction in the search space or even to infeasibility problem, on the other hand, a high order of the polynomial F leads to a larger space of research therefore a good performance of the resulting controller but the computational burden is also higher, so the choice should be moderated.

The unit step responses of both proposed GPC_RST and initial GPC_RST control system are shown in Fig. 4.a, Fig. 4.ba and b while the control signals produced by these control systems are shown in Fig. 4c. It is apparent that the proposed GPC_RST controller produces a unit step response without overshoot and guarantees a settling time of 2s as expected, furthermore, the error settling is within the desired limits (±1%), while the given bounds on the control signal are respected.

**Fig. 4.a:**
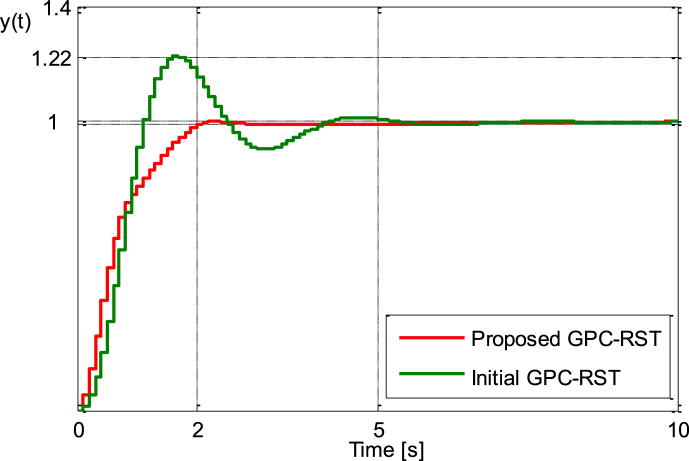
Step responses (Example 1).

**Fig. 4.b:**
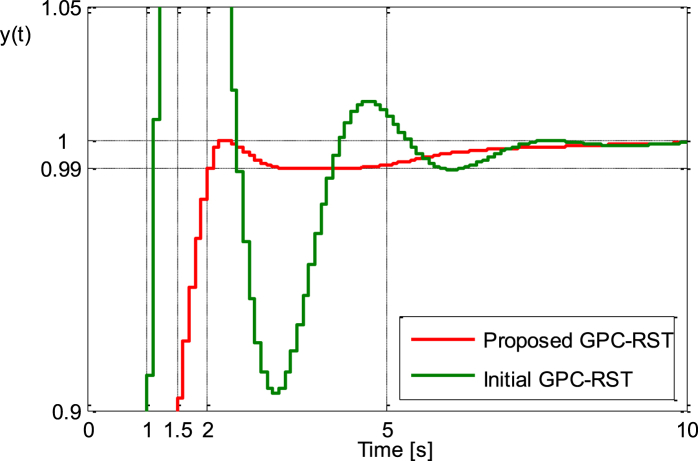
Step responses zoomed between [0.9 and 1.05] (Example 1).

**Fig. 4.c:**
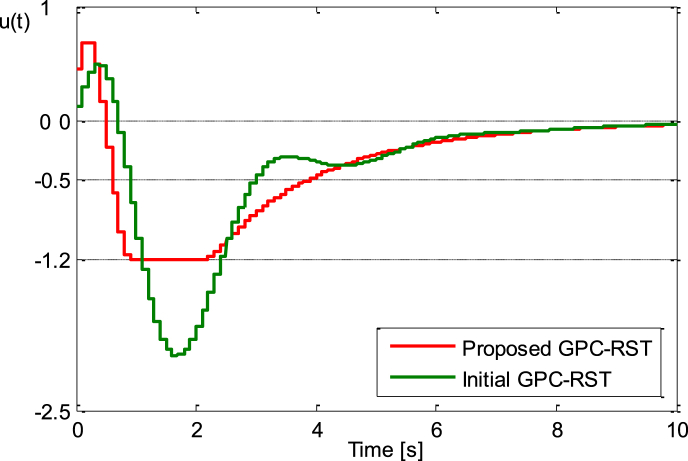
Control signals (Example 1).

### 4.2 Example 2: multi-lag process

Consider the following multi-lag process which was given in [25].

$$G\left(s\right)=\frac{1}{{\left(s+1\right)}^{5}}$$

The sampling period is chosen as ${\;T}_{s}=0.1s$. The equivalent discrete time model is

$$G\left(q^{-1}\right)=q^{-1}\frac{10^{-5}*\left(0.007+0.183q^{-1}+0.428q^{-2}+0.155q^{-3}+0.005q^{-4}\right)}{1{-4.524q}^{-1}+8.187q^{-2}-7.408q^{-3}+3.351q^{-4}-0.606q^{-5}}$$

The control objective is to let output response track a step reference from 0 to 1 as close as possible with short settling time and without overshoot. This goal is not reachable by the conventional GPC predictive control. Below, we will demonstrate that this is possible by applying the proposed methodology developed in section III.

Firstly, in order to perform a quick response, we select the following tuning parameters ${\;N}_{1}=1,N_{2}=15,N_{u}=1$ for the initial GPC_RST controller that a very high control effort is resulted with an overshoot of 112.8 % and very longer settling time about 7.4s,

By applying the developed method, we adopt the following conditions to get a good performance in terms of the settling time, settling error, control effort, and overshoot.

$$\text{Zero}\;\text{overshoot}\;\text{and}\;\text{zero}\;\text{undershoot}\;\to \;y_{1\;\max}=1,y_{1\;\min}=0$$

$$\text{Short}\;\text{settling}\;\text{time}\;\to \;N_{s}T_{s}=4s$$

$$\text{Minimal}\;\text{settling}\;\text{error}\;\to \;y_{2\;\max}=1,y_{2\;\min}=0.99$$

Controlsignalbounded→−5≤u(t)≤5

The research of the F parameter has been achieved with *40-*order polynomial and with $N_{t}=200$ samples equivalent to 10s.

This setting provide the results of Fig. 5.a, Fig. 5.b, Fig. 5.ca, b and c, where the results for the improved GPC_RST controller are compared to the ones obtained with the initial GPC_RST controller. It is easy to find that the proposed strategy can meet the given requirements simultaneously.

**Fig. 5.a:**
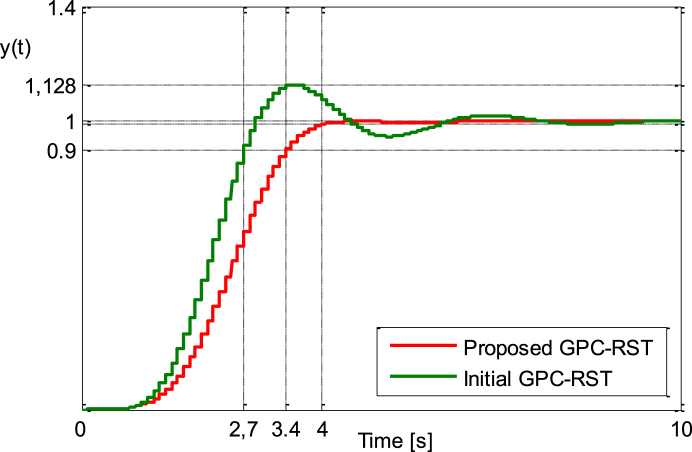
Step responses (Example 2).

**Fig. 5.b:**
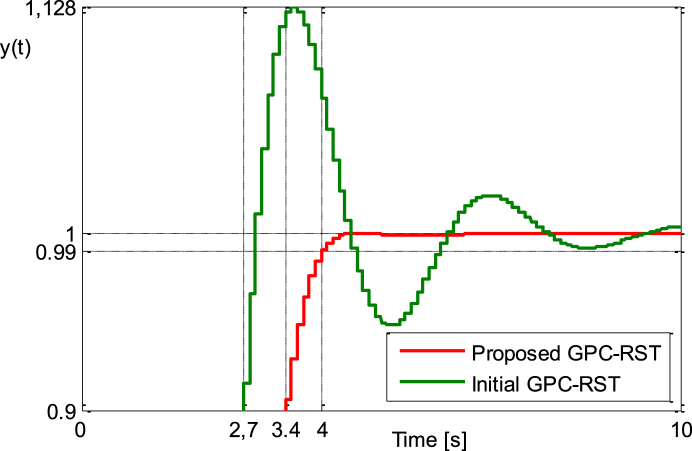
Step responses zoomed between [0.9 and 1.128] (Example 2).

**Fig. 5.c:**
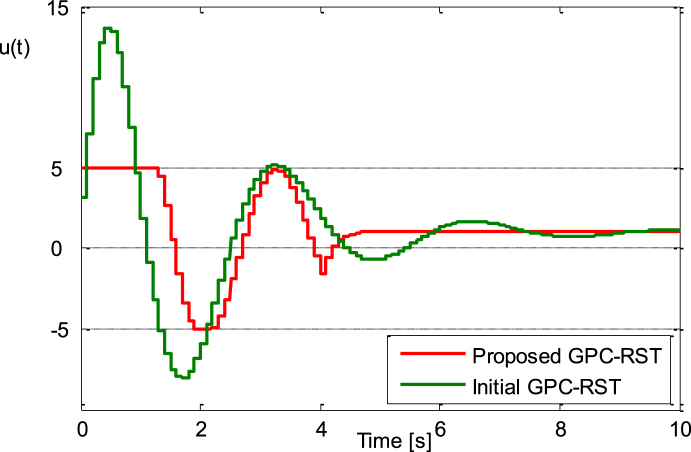
Control signals (Example 2).

From an overall perspective, the proposed method can accomplish a satisfactory transient response in terms of settling time, overshoot, and settling error, and this is achieved by a control signal having a smaller magnitude.

## 5 Conclusion

This work presents a new design technique for enhancing the tracking performance of the GPC_RST controller by using an additional polynomial in the feedforward path inside the standard GPC control system. The main contribution consists of an off-line improvement of the transient response indexes in terms of settling time, rising time, overshoot, settling error, and transient control signal. This is performed as a convex optimization problem of extra parameter solved with LP solver. Tests on two processes are carried out to verify the validity of the presented approach, and results prove the superiority of the developed GPC_RST controller.

## CRediT authorship contribution statement

**Khelifa Khelifi Otmane:** Writing – review & editing, Writing – original draft, Conceptualization. **Taieb Bessaad:** Supervision, Resources, Methodology.

## Data availability statement

No additional data was used for the research described in the paper.

## Declaration of competing interest

The authors declare that they have no known competing financial interests or personal relationships that could have appeared to influence the work reported in this paper.
